# High central venous saturation after cardiac surgery is associated with increased organ failure and long-term mortality: an observational cross-sectional study

**DOI:** 10.1186/s13054-015-0889-6

**Published:** 2015-04-16

**Authors:** Felix Balzer, Michael Sander, Mark Simon, Claudia Spies, Marit Habicher, Sascha Treskatsch, Viktor Mezger, Uwe Schirmer, Matthias Heringlake, Klaus-Dieter Wernecke, Herko Grubitzsch, Christian von Heymann

**Affiliations:** Department of Anesthesiology and Intensive Care Medicine, University Hospital Charité, Campus Charité Mitte/Campus Virchow Klinikum, Charité-Universitätsmedizin Berlin, Charitéplatz 1, 10098 Berlin Germany; Department of Anaesthesiology and Intensive Care Medicine, Jena University Hospital, Erlanger Allee 101, 07747 Jena, Germany; Department of Cardiovascular Surgery, University Hospital Charité, Campus Charité Mitte/Campus Virchow Klinikum, Charité-Universitätsmedizin Berlin, Charitéplatz 1, 10098 Berlin, Germany; Institute of Anaesthesiology Heart and Diabetes Center Nordrhein-Westfalen, University Clinic Ruhr-University Bochum, Georgstrasse 11, 32545 Bad Oeynhausen, Germany; Department of Anaesthesiology and Intensive Care Medicine, University of Lübeck, Ratzeburger Allee 160, 23538 Lübeck, Germany; SOSTANA GmbH, Wildensteiner Straße 27, 10318 Berlin, Germany

## Abstract

**Introduction:**

Central venous saturation (S_cv_O_2_) monitoring has been suggested to address the issue of adequate cardiocirculatory function in the context of cardiac surgery. The aim of this study was to determine the impact of low (L) (<60%), normal (N) (60%-80%), and high (H) (>80%) S_cv_O_2_ measured on intensive care unit (ICU) admission after cardiac surgery.

**Methods:**

We conducted a retrospective, cross-sectional, observational study at three ICUs of a university hospital department for anaesthesiology and intensive care. Electronic patient records of all adults who underwent cardiac surgery between 2006 and 2013 and available admission measurements of S_cv_O_2_ were examined. Patients were allocated to one of three groups according to first S_cv_O_2_ measurement after ICU admission: group L (<60%), group N (60%-80%), and group H (>80%). Primary end-points were in-hospital and 3-year follow-up survival.

**Results:**

Data from 4,447 patients were included in analysis. Low and high initial measurements of S_cv_O_2_ were associated with increased in-hospital mortality (L: 5.6%; N: 3.3%; H: 6.8%), 3-year follow-up mortality (L: 21.6%; N: 19.3%; H: 25.8%), incidence of post-operative haemodialysis (L: 11.5%; N: 7.8%; H: 15.3%), and prolonged hospital length of stay (L: 13 days, 9–22; N: 12 days, 9–19; H: 14 days, 9–21). After adjustment for possible confounding variables, an initial S_cv_O_2_ above 80% was associated with adjusted hazard ratios of 2.79 (95% confidence interval (CI) 1.565-4.964, *P* <0.001) for in-hospital survival and 1.31 (95% CI 1.033-1.672, *P* = 0.026) for 3-year follow-up survival.

**Conclusions:**

Patients with high S_cv_O_2_ were particularly affected by unfavourable outcomes. Advanced haemodynamic monitoring may help to identify patients with high S_cv_O_2_ who developed extraction dysfunction and to establish treatment algorithms to improve patient outcome in these patients.

**Electronic supplementary material:**

The online version of this article (doi:10.1186/s13054-015-0889-6) contains supplementary material, which is available to authorized users.

## Introduction

Postoperative mortality and morbidity of surgical patients are still unsolved puzzles for the clinician [1]. In a multicentre study, the rates of death for patients who underwent surgery varied substantially across hospitals. A recent European study confirmed these results [2,3]. This finding was taken up by initiatives to improve care for the surgical patient. Worldwide, routine care began including goal-directed optimization in the perioperative period to avoid hypoperfusion and secondary organ failure. According to recent meta-analyses, this strategy showed improved outcomes in the cohort of optimized patients [4]. Nevertheless, this approach will be successful only if the haemodynamic goal still holds.

Cardiac surgery has been shown to be a life-saving procedure in a selected group of patients with coronary heart disease and valve dysfunction [5]. However, this procedure carries a significant risk of death and major complications. In recent years, there has been substantial progress in perioperative intensive care management in decreasing the risk for unfavourable outcomes in this group of patients [6,7]. One major step forward was the adoption of early goal-directed, evidence-based haemodynamic treatment strategies in cardiac surgery patients [6]. Goal-directed administration of perioperative fluids has been shown to guarantee the best possible optimization of volume therapy in these patients [8]. Several single-centre studies and meta-analyses have shown improved outcomes by using additional haemodynamic parameters to adjust individual therapy in the perioperative setting [4] of general and cardiac surgery. In cardiac surgery patients in particular, cardiac dysfunction must be detected as early as possible to facilitate adequate therapy using positive inotropic drugs as well as fluids and vasodilators [8].

Central venous saturation (S_cv_O_2_) monitoring has been suggested as one goal to address the issue of adequate cardio-circulatory function in the context of cardiac surgery [6]. This parameter seems especially attractive as venous saturation is readily measurable without additional monitoring technology and is an excellent indicator of the match/mismatch between cardiac output, arterial oxygen saturation, and haemoglobin level as determinants of both oxygen delivery and oxygen consumption. Several studies in the cardiac-surgery patient population and in other surgical specialties have shown that low S_cv_O_2_ and mixed venous saturation (S_v_O_2_) are associated with unfavourable outcomes [9,10]. In addition, high venous saturation has been shown to be associated with increased complication rates in emergency room patients, intensive care unit (ICU) patients, and cardiac-surgery patients [11-13]. However, the results of these studies may be biased by the small number of patients. The aim of this retrospective cross-sectional study, therefore, was to investigate the impact of low, normal, and high S_cv_O_2_ on postoperative mortality and organ dysfunction in a large cardiac surgery ICU database that reported data from three distinct ICUs at our institution.

## Methods

With the written consent of the federal data protection officer and the hospital ethics commission (ethics committee - Charité - Universitätsmedizin Berlin, EA1/034/13), clinical routine data from all patients who underwent cardiac surgery between 2006 and 2013 were extracted from the two electronic patient data management systems at our hospital (COPRA System, Sasbachwalden, Germany, and SAP, Walldorf, Germany) into an anonymized study database. Individual patient consent was waived by the ethics commission. Cardiac surgery was defined as a documented procedure on valves or vessels in proximity to the heart or coronary vessels. Cardiopulmonary bypass and anaesthesia management were performed in accordance with the department’s standard operating procedures described in detail elsewhere [14]. Haemodynamic optimization was continuously accomplished in accordance with the German guidelines [6].

To be included in our study, patients had to be admitted postoperatively to one of our three ICUs serving postoperative cardiac surgery patients. Patients who were under the age of 18 by the time of surgery or who had a pulmonary artery catheter were excluded. Patients were grouped on the basis of first measurement of S_cv_O_2_ after ICU admission as low, normal, or high according to a normal range defined as 60% to 80%. S_cv_O_2_ in the ICU was determined by point-of-care blood gas analysis (ABL800 FLEX; Radiometer, Copenhagen, Denmark). Mortality, length of stay, time of ventilation, renal dysfunction, and days without organ failure were defined as outcome criteria. Information on long-term mortality, defined as 3 years after ICU admission, was acquired, with clearance from the federal data safety officer, by consulting the registry office in Berlin, Germany. Patients with shorter observation intervals (that is, less than 3 years between ICU admission and the date of inquiry at the registry office) were excluded in analyses of long-term mortality. Renal dysfunction was evaluated in accordance with the creatinine criteria of the KDIGO (Kidney Disease: Improving Global Outcomes) clinical practice guidelines for acute kidney injury [15]. Patients with pre-operative haemodialysis were excluded in analysis on renal outcome parameters. Days of organ failure were assessed in accordance with the criteria of the Sequential Organ Failure Assessment score with a tolerance of 1 point in each category (respiration: partial pressure of oxygen/fraction of inspired oxygen (PaO_2_/FiO_2_) of less than 300; circulation: use of epinephrine or norepinephrine; central nervous system: Glasgow Coma Scale score of less than 13; bilirubin of at least 2.0 mg/dL; kidneys: serum creatinine of at least 2.0 mg/dL).

To control for possible confounding variables, we assessed the risk of cardiac surgery by using the Age, Creatinine, and Ejection Fraction (ACEF) risk score by Ranucci *et al*. [16] and post-operative ICU admission scores—Acute Physiology And Chronic Health Evaluation II (APACHE II) and Therapeutic Intervention Scoring System (TISS)—in addition to basic patient characteristics. The design of this observational cohort study and the results are reported in accordance with the STROBE (Strengthening the Reporting of Observational Studies in Epidemiology) statement [17].

### Statistical analysis

Descriptive analyses and statistical testing were performed by using the R Project of Statistical Computing 3.0.1. When normal distribution was ruled out by using the Kolmogorov-Smirnov test, results were presented as median and interquartile range (IQR) or as mean ± standard deviation. Qualitative observations were characterized by numbers and percentages. Statistical significance among groups was analysed univariately with the exact non-parametric Kruskal-Wallis test and (pair-wise) with the exact Mann–Whitney *U* test. Exact chi-squared tests were used for qualitative data. Correlations were evaluated by using Spearman’s correlation coefficient. Survival was analysed by using Kaplan-Meier estimations and tested by the log-rank test between groups. Multivariate analysis tested for factors influencing survival. For that purpose, all pre-operative variables, ICU admission scores, and cumulative dosages (first 24 hours in the ICU) of inotropic drugs that showed significant *P* values in univariate Cox regression were included (besides the grouping) in multivariate Cox regression analyses with stepwise backwards selection. Clinical outcomes with respect to time were analysed by using a non-parametric analysis of longitudinal data in a two-factorial design (first factor: groups; second factor: time). A two-tailed *P* value of less than 0.05 was considered statistically significant. All tests should be understood as constituting explorative analysis; no adjustment for multiple testing has been made.

## Results

**Figure 1:**
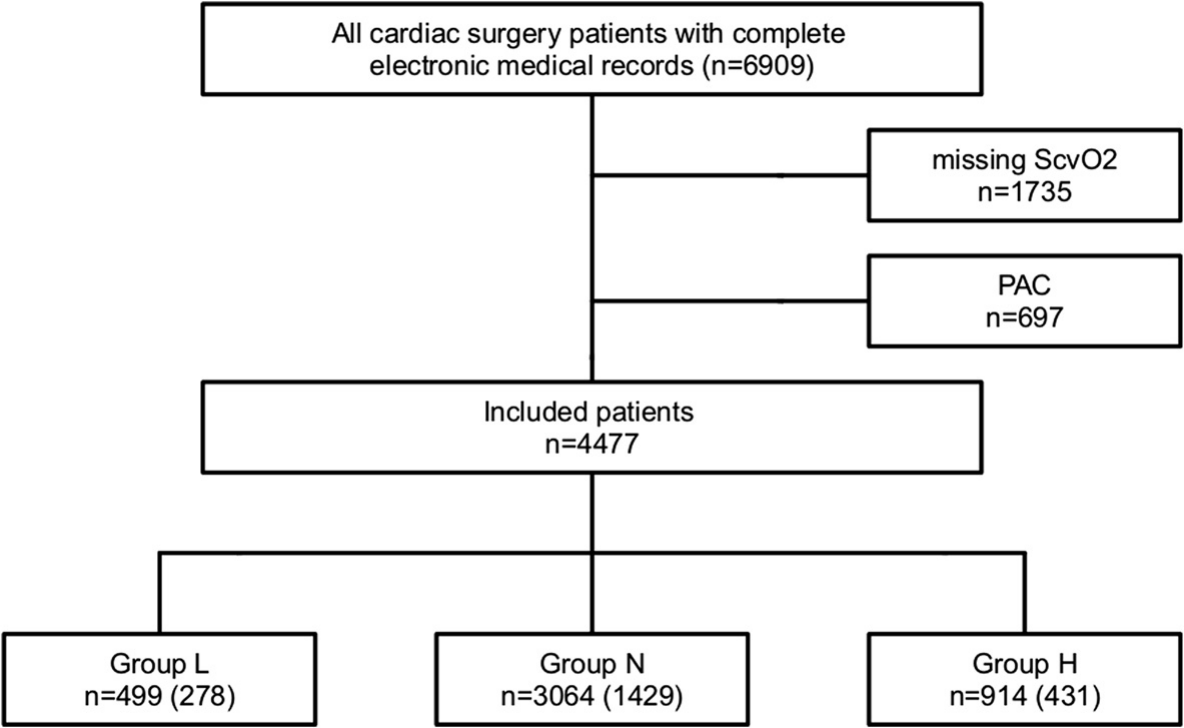
**CONSORT (Consolidated Standards Of Reporting Trials) diagram.** In the last three boxes containing the number of patients per group who were included in analyses, the parenthesis present the number of patients with complete 3-year follow-up data. PAC, pulmonary artery catheter; S_cv_O_2_, central venous saturation.

In this retrospective analysis, data from 6,909 patients who underwent cardiac surgery between January 2006 and December 2013 were analysed. There were no records for S_cv_O_2_ in 1,735 patients, and 697 patients received a pulmonary artery catheter. Both resulted in exclusion from the study, leaving a total of 4,477 patients for analysis. For sub-analysis on long-term survival, complete follow-up data were available for 2,138 patients (Figure 1). In all patients, median S_cv_O_2_ was 72.2 (IQR 65.5-78.5) with a standard deviation of 9.9 and was measured, on average, within 157 minutes (IQR 81–366) after admission to the ICU.

In 499 patients, S_cv_O_2_ was less than 60% (group L), in 3,064 patients S_cv_O_2_ was between 60 and 80% (group N), and in 914 patients S_cv_O_2_ was more than 80% (group H) at admission to the ICU after cardiac surgery.

Demographic and surgery-related data are shown in Table 1. Patients with low S_cv_O_2_ were slightly older than patients from the other two groups. Patients from group H had significantly lower body mass index compared with the other two groups. The predominant type of surgery was coronary artery bypass graft surgery in all three groups. Duration of anaesthesia and surgery did not differ significantly among groups. APACHE II and TISS scores were significantly higher in group H compared with reference group N; however, the slight difference (not more than 1 point) might not be considered clinically relevant. Patients from group H presented a significantly increased ACEF score. Patients from group H were significantly less frequently diagnosed with coronary heart disease compared with those from group N. Also, patients from group H were significantly more frequently diagnosed with atrial fibrillation and chronic renal insufficiency than patients from the reference group (Table 1).

**Table 1:** **Patient characteristics**

	**Group L**	**Group N**	**Group H**	**Number**
	**N = 499**	**N = 3,064**	**N = 914**	
**Basic data**				
Age, years	71.0* (64.0–75.0)	69.0 (61.0–75.0)	69.0 (61.0–75.0)	4,477
Sex: Female	149 (29.9%)	862 (28.1%)	264 (28.9%)	4,477
Body mass index	27.4 (24.5–30.6)	27.0 (24.4–30.4)	26.4* (23.7–29.6)	3,202
**Surgery**				
Type of surgery	*		*	4,477
CABG	290 (58.1%)	1821 (59.4%)	441 (48.2%)	
Valves	129 (25.9%)	891 (29.1%)	367 (40.2%)	
Both	80 (16.0%)	352 (11.5%)	106 (11.6%)	
Duration of anesthesia, minutes	290 (245–335)	290 (240–335)	285 (235–330)	3,344
Duration of surgery, minutes	202 (165–245)	200 (160–240)	190 (155–240)	3,594
Pre-op risk assessment (ACEF)	1.28 (1.14–1.54)	1.27 (1.12–154)	1.43* (1.20–1.98)	2,377
**Scores on ICU admission**				
APACHE II	18.0 (14.0–24.0)	18.0 (14.0–24.0)	19.0* (14.0–25.0)	4,008
TISS28	36.0 (33.0–39.0)	36.0 (33.0–39.0)	36.0* (33.0–40.0)	4,456
**Pre-existing medical conditions**				
Coronary heart disease	407 (81.6%)	2392 (78.1%)	662* (72.4%)	4,477
COPD	79 (15.8%)	526 (17.2%)	172 (18.8%)	4,477
Diabetes mellitus	208 (41.7%)	1,205 (39.3%)	369 (40.4%)	4,477
Peripheral vascular disease	97 (19.4%)	611 (19.9%)	197 (21.6%)	4,477
Atrial fibrillation	151 (30.3%)	859 (28.0%)	326* (35.7%)	4,477
Chronic renal insufficiency	118 (23.6%)	696 (22.7%)	309* (33.8%)	4,477

Values are presented as number (percentage) or as median (interquartile range). Significant group differences are displayed as asterisks in columns for groups L (low) and H (high) with reference to group N (normal). ACEF, Age, Creatinine, and Ejection Fraction; APACHE II, Acute Physiology And Chronic Health Evaluation II; CABG, coronary artery bypass graft; COPD, chronic obstructive pulmonary disease; ICU, intensive care unit; TISS28, Therapeutic Intervention Scoring System 28.

Patients from group H had the highest in-hospital mortality (6.8%), compared with 5.6% in the group with low S_cv_O_2_ and 3.3% in group N (Table 2). Three-year follow-up data were available for 3,517 out of 4,477 included patients. Of these, 1,379 patients were excluded because the interval between surgery and the retrospective data collection was less than 3 years. Results for 3-year follow-up mortality show a similar distribution among groups as in-hospital mortality with 19.3% for group N, 21.6% for group L, and 25.8% for group H. Lengths of hospital stay, ICU stay, and postoperative ventilation were significantly longer in patients from group H compared with patients from group N. Patients from group H also had the highest rate of renal dysfunction. In keeping with that finding, the incidence of postoperative need for renal replacement therapy (RRT) was 15.3% in patients with S_cv_O_2_ above 80% compared with 9.4% in group N and 13.2% in group L (Table 2). To rule out the time of admission as a confounder for prolonged ventilation, we compared the hours of admission as a binary variable (that is, 8 to 19 versus 20 to 27 hours). No significant difference was found among groups. Outcome parameters are reported separately with respect to the type of surgery that was performed (that is, coronary artery bypass graft, valves, or both) in Additional file 1: Tables S2a-c of the electronic supplement.

**Table 2:** **Selected outcome parameters**

	**Group L**	**Group N**	**Group H**	**Number**
	**N = 499**	**N = 3,064**	**N = 914**	
**General outcome measures**				
Mortality (in-hospital)	28* (5.6%)	102 (3.3%)	62* (6.8%)	4,477
Mortality (3-year follow-up)	60 (21.6%)	276 (19.3%)	111* (25.8%)	2,138
LOS (hospital), days	13.0* (9.00–22.0)	12.0 (9.00–19.0)	14.0* (9.00–21.0)	4,477
LOS (ICU), days	6.0* (4.0–9.0)	5.0 (3.0–8. 0)	6.0* (4.0–100)	4,477
Time of ventilation, hours	9.0 (6.0–16.0)	8.0 (5.0–15.0)	12.0* (7.0–21.0)	4,296
KDIGO	*		*	4,165
Stage 0	324 (70.4%)	2288 (79.4%)	589 (71.5%)	
Stage 1	75 (16.3%)	302 (10.5%)	86 (10.4%)	
Stage 2	9 (2.0%)	49 (1.7%)	20 (2.4%)	
Stage 3	52 (11.3%)	242 (8.4%)	129 (15.7%)	
KDIGO Stages 1-3	136* (29.6%)	593 (20.6%)	235* (28.5%)	4,165
Incidence of haemodialysis	56* (11.5%)	235 (7.82%)	130* (15.3%)	4,344
**Mean percentage of days in the ICU without organ failure**				
Respiration	58.0%*	64.1%	62.7%	4,477
Liver	98.3%	98.6%	97.8%	4,477
Circulation	48.3%*	55.2%	50.0%*	4,477
Central nervous system	80.1%	83.0%	80.5%	4,477
Kidneys	84.7%*	88.9%	84.4%*	4,344

Values are presented as number (percentage) or as median (interquartile range). Significant group differences are displayed as asterisks in columns for groups L (low) and H (high) with reference to group N (normal). Renal dysfunction was assessed in accordance with the KDIGO (Kidney Disease: Improving Global Outcomes) classification, calculating the change in serum creatinine from pre-op until postoperative day 7. Days of organ failure were calculated according to the criteria of the Sequential Organ Failure Assessment score. Respiration: partial pressure of oxygen/fraction of inspired oxygen (PaO_2_/FiO_2_) of less than 300; bilirubin of at least 2.0 mg/dL; circulation: use of epinephrine or norepinephrine; central nervous system: Glasgow Coma Scale score of less than 13; kidneys: serum creatinine of at least 2.0 mg/dL. Patients with pre-operative haemodialysis (n = 133) were excluded from analysis of renal outcome parameters. ICU, intensive care unit; LOS, length of stay.

**Figure 2:**
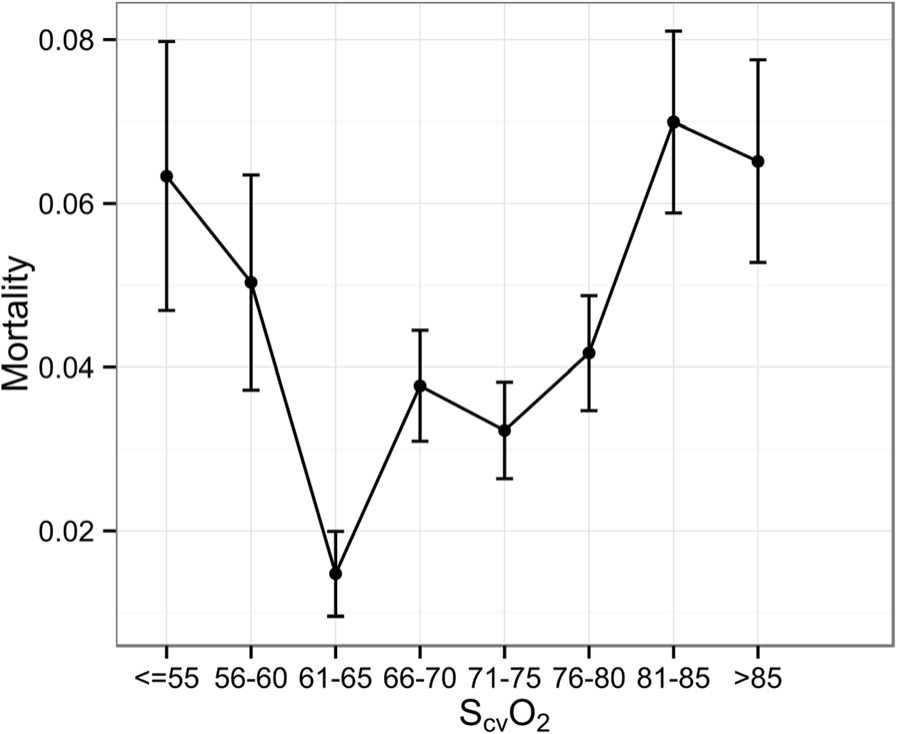
**In-hospital mortality for specified ranges of central venous saturation (S**_**cv**_ **O**_**2**_ **).** Mortality is highest for venous saturations below 60% and above 80% with a trough between 60% and 80%.

In Figure 2, in-hospital mortality in pre-specified subsets of initial S_cv_O_2_ measurements is shown. There is a U-shaped relationship between initial S_cv_O_2_ and mortality, showing highest mortality for patients with S_cv_O_2_ below 60% and above 80%.

In regard to the use of inotropic medication during the first 24 hours after surgery, patients from group H received epinephrine more frequently and received enoximone with respectively higher dosages (Table 3).

**Table 3:** **Inotropic medication on postoperative day one**

	**Group L**	**Group N**	**Group H**	**Number**
	**N = 499**	**N = 3,064**	**N = 914**	
**Norepinephrine**				
Frequency	275 (55.1%)	1,790 (58.4%)	683* (74.7%)	4,477
Dosage, mg/24 hours	1.17 (0.33–3.30)	1.11 (0.26–4.08)	2.20* (0.52–6.58)	2,748
**Epinephrine**				
Frequency	63 (12.6%)	427 (13.9%)	384* (42.0%)	4,477
Dosage, mg/24 hours	0.51* (0.25–2.66)	1.35 (0.43–3.23)	1.82* (0.64–4.13)	874
**Dobutamine**				
Frequency	317* (63.5%)	2,106 (68.7%)	486* (53.2%)	4,477
Dosage, mg/24 hours	227* (121–382)	190 (85.1–321)	134* (53.1–266)	2,909
**Enoximone**				
Frequency	84* (16.8%)	324 (10.6%)	229* (25.1%)	4,477
Dosage, mg/24 hours	173 (75.4–265)	185 (92.7–294)	222* (127–305)	637
**Levosimendan**				
Frequency	4 (0.80%)	23 (0.75%)	17* (1.86%)	4,477
Dosage, mg/24 hours	5.79 (3.55–8.11)	7.74 (2.85–9.66)	8.50 (5.37–9.38)	44

Values are presented as number (percentage) or as median (interquartile range). Significant group differences are displayed as asterisks in columns for groups L (low) and H (high) with reference to group N (normal). When a patient was discharged or was deceased during postoperative day 1, displayed dosages were normalized to 24 hours.

Patients of group H had significantly elevated levels of lactate, blood glucose, leukocyte count, and procalcitonin (PCT) as inflammation markers in the immediate period after surgery (Table 4). Creatinine, international normalized ratio, and creatine kinase MB also showed elevated levels in patients of group H. The calculated oxygen extraction rate ranged from 0.15 (IQR 0.12-0.17) in group H to 0.42 (IQR 0.41-0.46) in group L (*P*_groups_ <0.001 in non-parametric longitudinal analyses). For the postoperative course of S_cv_O_2_ and cardiopulmonary basic monitoring, please see Additional file 1: Table S1 in the electronic supplement.

**Table 4:** **Postoperative laboratory data**

	**Group L**	**Group N**	**Group H**	**Number**
	**N = 499**	**N = 3,064**	**N = 914**	
**Lactate, mg/dL**			*	
0–6 hours	15.0 (10.0-21.0)	15.0 (10.0-22.0)	22.0* (12.0-50.8)	4,188
6–12 hours	14.0 (10.0-20.0)	14.0 (10.0-21.0)	16.0* (10.0-26.0)	4,254
12–18 hours	11.0 (9.00-15.0)	11.0 (9.00-16.0)	12.0* (9.00-17.0)	4,274
18–24 hours	12.0 (9.00-16.0)	12.0 (9.00-16.0)	12.0 (9.00-16.0)	4,335
**Blood glucose, mg/dL**	*			
0–6 hours	142* (123–164)	148 (129–169)	152* (126–184)	4,188
6–12 hours	132* (117–149)	135 (119–153)	137* (119–158)	4,254
12–18 hours	128 (112–146)	131 (116–146)	129 (112–148)	4,254
18–24 hours	136 (118–156)	137 (120–157)	135 (118–158)	4,335
**Haemoglobin, g/dL**	*			
POD 0	10.0* (9.30-10.8)	10.3 (9.50-11.1)	10.3 (9.50-11.3)	4,350
POD 1	9.70 (9.10-10.4)	9.70 (9.00-10.5)	9.80 (9.10-10.6)	4,477
POD 2	9.60 (9.10-10.4)	9.70 (9.00-10.4)	9.70 (9.10-10.4)	4,477
POD 3	9.70 (9.20-10.5)	9.80 (9.20-10.6)	9.80 (9.20-10.6)	4,477
**Leukocytes, /nL**			*	
POD 0	12.0 (9.48-15.4)	12.4 (9.75-15.5)	13.2* (10.1-16.8)	4,171
POD 1	13.1 (10.7-16.6)	13.8 (11.0-16.7)	14.4* (11.3-17.8)	4,413
POD 2	13.6 (11.0-16.9)	14.0 (11.2-17.1)	14.9* (11.6-18.6)	4,439
POD 3	11.9 (9.27-15.0)	11.6 (9.36-14.4)	12.0* (9.50-15.6)	4,446
**Procalcitonin, μg/L**			*	
POD 0	0.48 (0.48-0.49)	0.58 (0.14-1.41)	1.70 (0.27-5.87)	22
POD 1	1.63 (0.39-3.98)	1.80 (0.56-5.28)	4.19* (1.62-11.8)	194
POD 2	1.06 (0.34-4.90)	1.52 (0.40-5.45)	4.37* (1.50-11.9)	562
POD 3	1.00 (0.34-3.27)	0.90 (0.30-3.57)	3.05* (0.80-8.45)	979
**Alanine transaminase, U/L**			*	
POD 0	22.0 (16.0-31.0)	22.0 (16.0-33.0)	23.0 (16.0-35.0)	3,999
POD 1	24.0 (17.0-35.0)	23.0 (17.0-35.0)	24.0 (17.0-38.0)	4,336
POD 2	24.0 (18.0-38.0)	24.0 (17.0-35.0)	25.0* (18.0-39.0)	4,389
POD 3	25.5 (18.0-39.0)	24.0 (17.0-36.0)	25.0 (18.0-40.0)	4,401
**Creatinine, mg/dL**	*		*	
Last preop	1.02* (0.86-1.20)	0.98 (0.83-1.18)	1.08* (0.88-1.35)	4,322
POD 1	1.14* (0.91-1.52)	1.06 (0.86-1.37)	1.19* (0.93-1.73)	4,412
POD 2	1.15* (0.90-1.55)	1.04 (0.82-1.42)	1.20* (0.90-1.77)	4,442
POD 3	1.11* (0.85-1.48)	1.00 (0.80-1.37)	1.16* (0.86-1.70)	4,449
**International normalized ratio**	*		*	
POD 0	1.31 (1.22-1.43)	1.30 (1.22-1.40)	1.33* (1.23-1.45)	4,153
POD 1	1.27 (1.19-1.37)	1.26 (1.18-1.35)	1.28* (1.20-1.39)	4,412
POD 2	1.26* (1.19-1.37)	1.25 (1.18-1.35)	1.27* (1.19-1.39)	4,437
POD 3	1.24* (1.15-1.34)	1.21 (1.13-1.31)	1.23* (1.14-1.35)	4,445
**Creatine kinase MB, U/L**	*		*	
POD 0	38.0* (27.0-55.5)	34.0 (26.0-52.0)	38.0* (26.0-61.0)	3,913
POD 1	33.0* (22.0-56.0)	31.0 (22.0-51.0)	33.0* (22.0-58.0)	4,191
POD 2	27.0* (19.0-46.0)	24.0 (17.0-39.0)	25.0 (17.0-43.0)	4,222
POD 3	23.5* (16.0-38.0)	21.0 (15.0-33.0)	21.0 (15.0-35.0)	4,233

Values are presented as median (interquartile range). Significant group differences are displayed as asterisks in columns for groups L (low) and H (high) with reference to group N (normal). Significance per category (for example, lactate) with respect to the given groups over time was assessed by using a non-parametric analysis of longitudinal data in a two-factorial design. POD, postoperative day.

**Figure 3:**
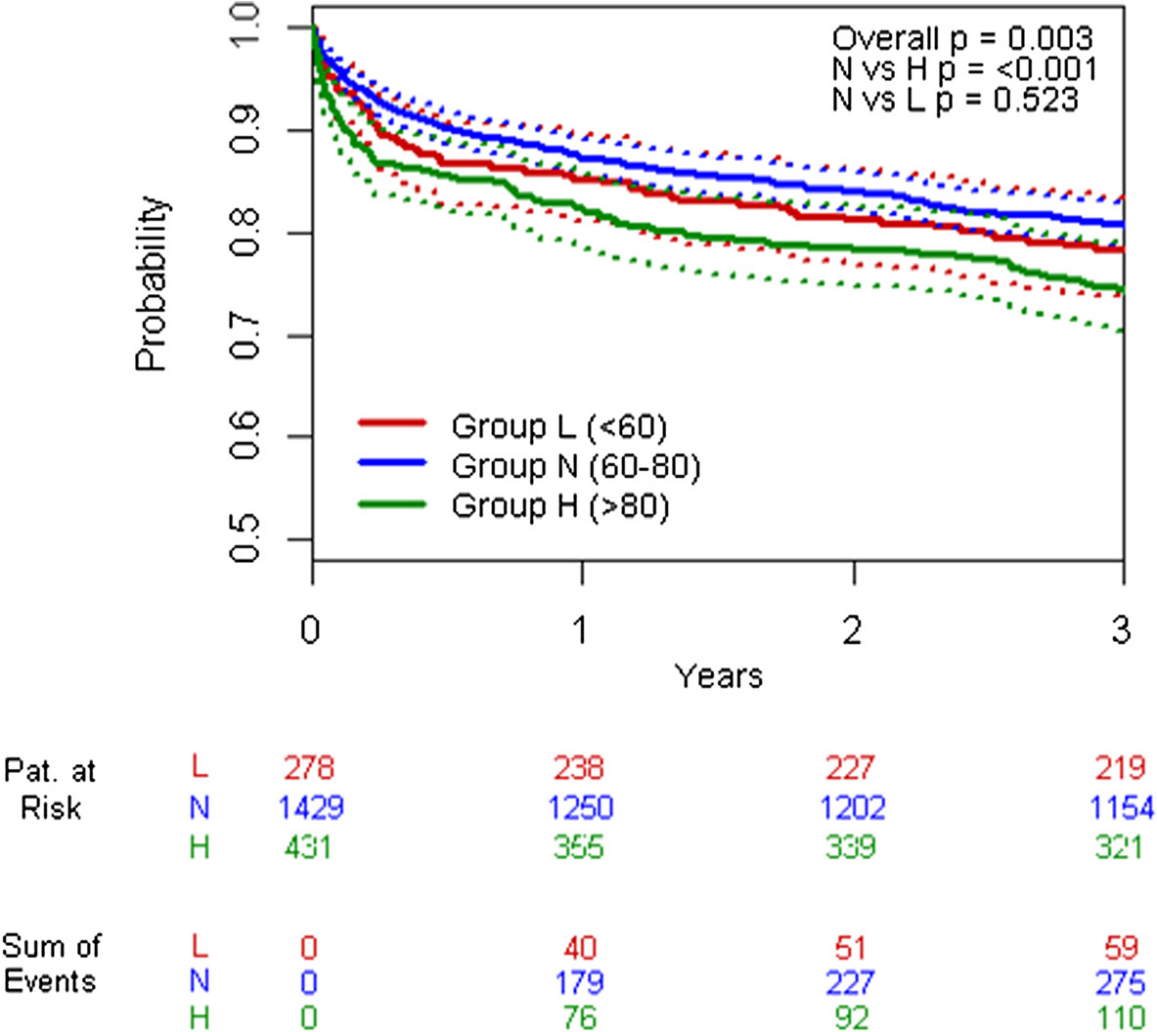
**Kaplan-Meier curves for long-term survival (3-year follow-up) of groups L (S**_**cv**_ **O**_**2**_ **< 60%), N (60% ≤ S**_**cv**_ **O**_**2**_ **≤ 80%), and H (S**_**cv**_ **O**_**2**_ **> 80%).** Only patients with a minimal observation period of 3 years were included. Patients from groups N and H differed significantly in pair-wise testing (log-rank test: *P* <0.001). H, high; L, low; N, normal; S_cv_O_2_, central venous saturation.

To evaluate multivariately the association of S_cv_O_2_ with survival, all pre-operative variables, ICU admission scores, and cumulative dosages (first 24 hours in the ICU) of inotropic drugs that showed significant *P* values in univariate Cox regression were included (besides grouping) in multivariate Cox regression analyses (Table 5). Compared with reference group N, patients from group H had an almost threefold risk of dying in hospital (hazard ratio 2.79, 95% confidence interval (CI) 1.565-4.964, *P* <0.001). The hazard ratio of group H (compared with N) for 3-year follow-up survival was 1.31 (95% CI 1.033-1.672, *P* = 0.026). When the incidence of pre-operative haemodialysis was included in the regression model as additional explanatory variable, the previously calculated hazard ratios did not change substantially. However, the regression model’s quality decreased. Kaplan-Meier curves for 3-year follow-up survival are shown in Figure 3. To investigate whether extraction dysfunction (that is, high S_cv_O_2_) could be predicted by a patient’s comorbidities, we developed another regression model. However, no significant effect was found (*P* >0.05, data not shown).

**Table 5:** **Multivariate Cox regression analysis for short- and long-term survival**

	**Cox regression for in-hospital survival**		**Cox regression for 3-year follow-up survival**	
	**HR unadjusted**	**HR adjusted**	**HR unadjusted**	**HR adjusted**
Group L, S_cv_O_2_ < 60	1.377 (0.905–2.096),	2.124 (0.985–4.582),	1.126 (0.911–1.392),	1.209 (0.893–1.638),
	*P* = 0.135	*P* = 0.055	*P* = 0272	*P* = 0.220
Group H, S_cv_O_2_ > 80	1.709 (1.244–2.348),	2.787 (1.565–4.964),	1.462 (1.244–1.718),	1.314 (1.033–1.672),
	*P* = 0.001	*P* <0.001	*P* <0.001	*P* = 0.026
Age in years	1.039 (1.023–1.056),	1.096 (1.056–1.138),	1.050 (1.041–1.058),	1.044 (1.029–1.059),
	*P* <0.001	*P* <0.001	*P* <0.001	*P* <0.001
Type of surgery: valves	1.480 (1.083–2.022),	1.858 (0.991–3.458),	1.596 (1.369–1.860),	1.438 (1.124–1.840),
	*P* = 0.014	*P* = 0.054	*P* <0.001	*P* = 0.004
Type of surgery: CABG + valves	1.469 (0.978–2.207),	1.747 (0.846–3.608),	1.835 (1.519–2.218),	1.281 (0.961–1.703),
	*P* = 0.064	*P* = 0.132	*P* <0.001	*P* = 0.088
Duration of surgery, minutes			1.003 (1.002–1.004),	1.002 (1.001–1.004),
			*P* <0.001	*P* = 0.010
Pre–operative ACEF score			2.609 (2.232–3.050),	1.379 (1.124–1.693),
			*P* <0.001	*P* = 0.002
APACHE II score on ICU admission			1.049 (1.039–1.060),	1.030 (1.014–1.047),
			*P* <0.001	*P* <0.001
TISS score on ICU admission	1.088 (1.069–1.107),	1.100 (1.060–1.142),	1.056 (1.045–1.068),	1.028 (1.009–1.047),
	*P* <0.001	*P* <0.001	*P* <0.001	*P* = 0.004
Atrial fibrillation			2.147 (1.869–2.466),	1.674 (1.340–2.091),
			*P* <0.001	*P* <0.001
COPD			1.798 (1.538–2.102),	1.497 (1.173–1.910),
			*P* <0.001	*P* = 0.001
Diabetes mellitus	1.440 (1.070–1.939),	2.076 (1.173–3.675),	1.735 (1.512–1.990),	1.405 (1.137–1.736),
	*P* = 0.016	*P* = 0.012	*P* <0.001	*P* = 0.002
Peripheral vascular disease	1.485 (1.100–2.006),	1.835 (1.064–3.164),	1.865 (1.601–2.174),	1.733 (1.364–2.201),
	*P* = 0.010	*P* = 0.029	*P* <0.001	*P* <0.001
Dobutamine, cumulative dosage mg/24 hours			0.999 (0.998–0.999),	0.999 (0.999–1.000),
			*P* <0.001	*P* = 0.098
Enoximone, cumulative dosage mg/24 hours			1.000 (1.000–1.000),	0.999 (0.998–1.000),
			*P* = 0.002	*P* = 0.144
Norepinephrine, cumulative dosage mg/24 hours	1.014 (1.012–1.017),	1.018 (1.011–1.024),	1.015 (1.012–1.017),	1.016 (1.011–1.021),
	*P* <0.001	*P* <0.001	*P* <0.001	*P* <0.001

Values are presented as median (interquartile range). Hazard ratios (HRs) are adjusted for all pre-operative variables, intensive care unit (ICU) admission scores, and cumulative dosages (first 24 hours in ICU) of inotropic drugs that were significant in univariate Cox regression. Variables have been selected by using stepwise backward selection. Reference for type of surgery: coronary artery bypass graft (CABG); for grouping variable: group N (60 ≤ S_cv_O_2_ ≤ 80). ACEF, Age, Creatinine, and Ejection Fraction; APACHE II, Acute Physiology And Chronic Health Evaluation II; COPD, chronic obstructive pulmonary disease; H, high; L, low; S_cv_O_2_, central venous saturation; TISS, Therapeutic Intervention Scoring System.

## Discussion

In this study, we report haemodynamic and outcome data from a large database including data from three different ICUs serving postoperative cardiac surgery patients. The most important findings of this study were that patients with a presumed ‘safe’ initial S_cv_O_2_ above 80% had the highest in-hospital mortality and the highest mortality in 3-year follow-up. Additionally, these patients showed increased length of mechanical ventilation, a higher incidence of renal dysfunction including increased rate of RRT, evidence of severe postoperative inflammation indicated by increased leukocyte counts and PCT levels, and prolonged lengths of ICU and hospital stay compared with patients with a ‘normal’ S_cv_O_2_ (60% to 80%). In the multivariate analysis, patients from group H had an almost threefold risk of dying in hospital and a 1.3 ratio for 3-year follow-up survival compared with group N.

Patients in the high S_cv_O_2_ group differed from the other S_cv_O_2_ groups with respect to the type of surgery and the pre-operative ACEF score and more frequently received inotropic and vasoactive drugs. Also, the patients in the high S_cv_O_2_ group had more frequent atrial fibrillation as a complication diagnosis. Thus, it was (per se) not astonishing that these patients also had a higher morbidity and mortality. Based on the classic pathophysiological concept, low levels of S_cv_O_2_ or S_v_O_2_ are reflective of tissue hypoperfusion and associated with higher complication rates [18]. Our finding that patients with a seemingly ‘safe’ S_cv_O_2_ above 80% showed even worse outcome is in clear contrast to previous studies.

Pre-existing chronic haemodialysis with arterial-venous shunts is known to lead to increased venous saturation. Therefore, a possible explanation for unfavourable outcome in group H could be this bias as patients with chronic renal failure are known to have an inferior outcome after surgery, including cardiac surgery. However, for this study cohort, this is not the explanation for the observed outcomes in group H as including incidence of pre-operative haemodialysis with arterial-venous shunts did not result in substantial changes of previously calculated hazard ratios.

Interestingly, the high S_cv_O_2_ group also presented with significantly increased plasma lactate levels. It is well accepted that increased lactate levels are associated with increased mortality after cardiac surgery [19]. Furthermore, it is of note that a recent retrospective study revealed a link between elevated lactate levels during normal or even high S_cv_O_2_ and unfavourable outcome [20]. In that study with 629 patients, elevated lactate levels in the setting of a normal S_cv_O_2_ were associated with significantly higher incidence of major complications. However, high lactate levels not only may be reflective of tissue hypoperfusion (that is, anaerobic metabolism leading to lactic acidosis type A) but also may be induced by factors stimulating glycogenolysis, glycolysis, and hyperglycemia (that is, lactic acidosis type B). It is of note that epinephrine is an important trigger for the latter effects [21] and that the patients in the high S_cv_O_2_ group more frequently received norepinephrine and epinephrine than the normal and the low S_cv_O_2_ groups, suggesting that the hyperlactatemia may be, at least in part, an effect of catecholamine treatment.

Additionally, as we found a significant higher occurrence of epinephrine application in group H, it cannot be ruled out that the use of epinephrine aggravates the shunting effects in postoperative cardiac surgery patients. If so, the question of whether epinephrine is what we are aiming for or just creates good numbers in the charts (for example, higher cardiac index and higher mean arterial pressure) clearly arises.

Totaro and Raper [22] have shown that 30% of patients receiving epinephrine after cardiac surgery developed lactic acidosis but that plasma lactate remained unchanged in patients who received norepinephrine. Comparably, small observational studies and case reports have shown an association between epinephrine use and hyperlactatemia [23]. A small randomized trial in cardiac surgery patients with postoperative low-cardiac output syndrome revealed significantly increased glucose and lactate levels and an increased lactate-pyruvate ratio in patients who received epinephrine in comparison with milrinone treatment, suggesting that epinephrine not only stimulated glycogenesis but also led to a situation with tissue malperfusion and anaerobic metabolism despite improved haemodynamics [24]. In line with this, Levy *et al*. [25] have shown that muscle tissue is a major source of lactate in patients with sepsis during treatment with epinephrine. The authors showed that patients with septic shock had increased skeletal muscle lactate production and that these metabolic alterations could be blocked by the Na^+^/K^+^-ATPase blocker ouabain, suggesting that the increased lactate levels were of a metabolic nature. Nonetheless, the lower oxygen extraction in the high S_cv_O_2_ group clearly suggests that—besides the metabolic effects of the infused catecholamines—tissue hypoperfusion may be another important component explaining the significantly increased rate of organ dysfunction and the higher mortality.

The concept of venous saturation monitoring to guide haemodynamic therapy is neither new nor peculiar. The first reports that used venous saturation monitoring to guide therapy in critically ill patients date back to the last century [26]. Accordingly, several reviews on this topic have been published in recent years [8,9] and concluded that venous saturation measurements may be used to monitor the adequacy of the circulation and to guide haemodynamic therapy in the perioperative period [4]. Venous saturation depends on the balance between oxygen delivery with its covariates—arterial oxygen saturation, cardiac output, and haemoglobin level—and oxygen consumption. With some limitations, S_cv_O_2_ can be used as a substitute for S_v_O_2_ [8,10]. In clinical practice, S_cv_O_2_ is often measured as the use of pulmonary arterial catheters in the context of critical illness; surgery has declined over the last decade [27].

Studies using venous saturation as a goal to optimize patients in the ICU reported beneficial results. In the landmark study by Rivers *et al*., performed in patients with severe sepsis and septic shock, this concept has been shown to decrease mortality in the patient group undergoing a treatment algorithm aiming for an S_cv_O_2_ of at least 70% [28]. Another study in a cardiac surgery patient population reported a decreased overall complication rate in patients optimized to an S_v_O_2_ of at least 70% and lactate below 2.0 mmol/L during ICU treatment after surgery [29]. In that study, patients of the protocol group had a shorter length of stay in the hospital and overall morbidity was lower by the time of hospital discharge.

In older as well as in recent studies, low S_cv_O_2_ and S_v_O_2_ were linked to unfavourable outcomes [11-13,30]. Thus, our finding of unfavourable outcomes in patients with S_cv_O_2_ below 60% is in line with previous reports. A study by Perz *et al*. investigated venous saturation measurements after cardiac surgery [13], indicating that an S_cv_O_2_ below 60.8% in patients undergoing cardiac surgery was associated with an unfavourable outcome.

However, optimizing venous saturation above a certain cutoff may carry some risks that lie within the physiology of venous saturations. Our finding of increased mortality and complication rate in patients with S_cv_O_2_ above 80% accords with smaller reports from critically ill patients and from patients after cardiac surgery.

Pope *et al*. reported data from 619 emergency department (ED) patients. In regard to the maximum S_cv_O_2_ during treatment in the ED, mortality rates of both the hypoxia group (40%) and the hyperoxia group (34%) were significantly higher than the rate of mortality in the normoxia group (21%) [11]. Therefore, in line with our results in cardiac surgery patients, central venous hyperoxia in ED patients with suspected sepsis seems to be associated with unfavourable outcomes as well. Yet another study provided evidence that hyperoxia in patients with sepsis is linked with unfavourable outcomes [12]. That study retrospectively analysed data from all ICU admissions of patients with septic shock and reported the maximum S_cv_O_2_ within the first 72 hours after the onset of shock. In conclusion, high levels of S_cv_O_2_ (more than 80%) in the first 72 hours after resuscitating patients in septic shock were associated with increased mortality which might reflect an impaired use of oxygen as evidenced by increased lactate levels [12].

### Limitations

Owing to the retrospective nature of our data, it is possible only to report associations and not causality. Therefore, we can only speculate about possible mechanisms leading to the increased complication and mortality rate in the group with high venous saturation measurements after cardiac surgery. Also, we cannot exclude that changes in management between 2006 and 2013 contributed to this finding. One further point of concern might be that S_cv_O_2_ measurements after admission to the ICU were missing in about one fourth of the total population. However, this seems negligible as baseline characteristics of all patients undergoing cardiac surgery regardless of effectuated S_cv_O_2_ measurements did not differ statistically (data not shown).

Nevertheless, we believe that it is important to provide evidence that high venous saturation measurements should not be misinterpreted as safe but should be considered to be associated with increased mortality and complication rates. Additionally, despite the increasing use of S_cv_O_2_ in clinical practice, several lines of evidence point out that, at least during states of increased oxygen extraction [10,20], the difference between S_cv_O_2_ and S_v_O_2_ may increase. In these situations, S_cv_O_2_ levels do not reliably reflect the ‘true’ systemic balance between oxygen delivery and demand. Nonetheless, with respect to the frequent use of S_cv_O_2_ in routine practice [27] and current recommendations for the use of S_cv_O_2_ for titrating haemodynamic therapy in cardiac surgery patients [6], the findings of the present study have relevant practical implications.

## Conclusions

We demonstrated that patients after cardiac surgery presenting with an initial S_cv_O_2_ higher than 80% on ICU admission—which might be considered safe or even optimal—had the highest in-hospital mortality and highest mortality in 3-year follow-up. This group also had increased lengths of ICU and hospital stay, increased mechanical ventilation after surgery, and increased rates of postoperative renal dysfunction and failure. Increased inflammation markers in patients with an initial S_cv_O_2_ higher than 80% might point to a postoperative ‘sepsis-like’ syndrome with oxygen extraction dysfunction. Therefore, it seems indicated that additional advanced haemodynamic monitoring may help to identify patients with high S_cv_O_2_ who developed extraction dysfunction and to establish treatment algorithms in order to improve outcome.

## Key messages

- In this study, we describe a statistically significant association between an initial S_cv_O_2_ higher than 80% on ICU admission after cardiac surgery and an increased in-hospital and 3-year follow-up mortality.
- Additional haemodynamic monitoring may help to identify patients with high S_cv_O_2_ who developed oxygen extraction dysfunction and to establish treatment algorithms in these patients to improve outcome.

## Additional file

Additional file 1:
**Supplementary tables for results section.**

## Abbreviations

ACEF: Age, Creatinine, and Ejection Fraction
APACHE II: Acute Physiology And Chronic Health Evaluation II
CI: confidence interval
ED: emergency department
H: high
ICU: intensive care unit
IQR: interquartile range
L: low
N: normal
PCT: procalcitonin
RRT: renal replacement therapy
S_cv_O_2_: central venous saturation
S_v_O_2_: mixed venous saturation
TISS: Therapeutic Intervention Scoring System
