# Supplementary material for: High central venous saturation after cardiac surgery is associated with increased organ failure and long-term mortality: an observational cross-sectional study
Source: Crit Care. 2015 Apr 16;19(1):168. doi: 10.1186/s13054-015-0889-6 (PMC4415351; doi:10.1186/s13054-015-0889-6)
Supplement: Additional file 1: — Supplementary tables for results section. [file 13054_2015_889_MOESM1_ESM.docx]

***Tables for electronic supplement***

Table S1: Postoperative course of ScvO2 and cardiopulmonary basic monitoring

|  | Group L | Group N | Group H |
| --- | --- | --- | --- |
|  | N=499 | N=3064 | N=914 |
| **ScvO2 [%]** |  |  |  |
| 0-6h | 57.0* [53.8;59.2] | 71.1 [66.5;75.3] | 82.6* [80.5;85.7] |
| 6-12h | 58.4*[54.8;64.4] | 68.9 [64.1;73.7] | 80.5* [71.5;83.9] |
| 12-18h | 59.8*[56.0;67.2] | 67.9 [63.2;72.7] | 75.5* [69.0;82.3] |
| 18-24h | 61.5* [56.2;68.3] | 67.7 [63.1;72.4] | 73.2* [67.3;80.8] |
| **Heart rate / min** |  |  |  |
| 0-6h | 79.3 [69.7;89.9] | 80.0 [69.9;90.5] | 82.4* [71.6;92.9] |
| 6-12h | 79.8 [71.0;90.4] | 79.3 [70.5;88.9] | 78.3 [69.8;89.7] |
| 12-18h | 80.1 [70.7;90.5] | 80.3 [71.3;89.2] | 80.0 [70.0;89.4] |
| 18-24h | 81.6 [72.0;90.3] | 80.5 [72.0;89.3] | 80.1 [71.3;90.3] |
| **Mean arterial pressure [mmHg]** |  |  |  |
| 0-6h | 71.7 [66.3;77.7] | 71.2 [65.7;77.3] | 70.7* [65.3;75.8] |
| 6-12h | 71.8 [66.3;77.4] | 71.7 [66.5;77.6] | 70.4*[65.2;75.7] |
| 12-18h | 72.2 [67.3;78.5] | 71.9 [66.7;78.2] | 71.1* [65.5;77.1] |
| 18-24h | 71.8 [65.2;77.7] | 72.0 [66.1;78.2] | 70.8*[65.3;77.0] |
| **SaO2 [%]** |  |  |  |
| 0-6h | 98.8 [97.3;100] | 98.8 [97.2;100] | 99.0* [97.6;100] |
| 6-12h | 98.0 [96.4;99.6] | 98.1 [96.5;99.5] | 98.5* [97.0;100] |
| 12-18h | 98.0 [96.1;99.3] | 97.9 [96.0;99.3] | 98.2* [96.6;99.6] |
| 18-24h | 97.7 [96.0;99.1] | 97.6 [95.8;99.0] | 98.0* [96.4;99.4] |

Significant group differences are displayed as asterisks in columns for groups L and H with reference to group N.

Table S2a: Selected outcome parameters – CABG only

|  | Group L | Group N | Group H |
| --- | --- | --- | --- |
|  | N=290 | N=1821 | N=441 |
| **General outcome measures:** | | | |
| Mortality (in-hospital) | 13 (4.48%) | 46 (2.53%) | 21 (4.76%) |
| Mortality (three-year follow-up) | 26 (16.8%) | 124 (15.1%) | 39 (18.8%) |
| LOS (Hospital) [d] | 12.0* [9.00;20.0] | 11.0 [8.00;16.0] | 12.0* [9.00;19.0] |
| LOS (ICU) [d] | 5.00* [4.00;8.00] | 5.00 [3.00;7.00] | 6.00* [4.00;9.00] |
| Time of ventilation [h] | 9.00 [6.00;15.0] | 8.00 [5.00;13.0] | 10.0* [6.00;19.0] |
| KDIGO: | * |  | * |
| Stage 0 | 190 (72.8%) | 1412 (82.8%) | 310 (78.9%) |
| Stage 1 | 45 (17.2%) | 165 (9.67%) | 35 (8.91%) |
| Stage 2 | 5 (1.92%) | 24 (1.41%) | 3 (0.76%) |
| Stage 3 | 21 (8.05%) | 105 (6.15%) | 45 (11.5%) |
| KDIGO 0 vs. 1-3 | 71* (27.2%) | 294 (17.2%) | 83 (21.1%) |
| Incidence of hemodialysis | 25 (8.80%) | 101 (5.62%) | 47* (11.4%) |
| **Mean percentage of days on ICU without organ failure:** | | | |
| Respiration | 59.0% | 64.7% | 63.7% |
| Liver | 98.7% | 99.3% | 98.4% |
| Circulation | 49.3% | 53.8% | 48.7% |
| Central nervous system | 82.5% | 84.8% | 83.5% |
| Kidneys | 86.1%* | 90.8% | 87.5% |

Significant group differences are displayed as asterisks in columns for groups L and H with reference to group N. Renal dysfunction was assessed according to the KDIGO classification, calculating the change in serum creatinine from pre-op until POD7. Days of organ failure were calculated according to the criteria of the Sequential Organ Failure Assessment score (SOFA). Respiration: PaO2/FiO2<300; Bilirubin: ≥ 2.0 mg/dl; Circulation: Use of epinephrine or norepinephrine; Central nervous system: GCS<13; Kidneys: Serum creatinine ≥ 2.0 mg/dl.

Table S2b: Selected outcome parameters – Valves only

|  | Group L | Group N | Group H |
| --- | --- | --- | --- |
|  | N=129 | N=891 | N=367 |
| **General outcome measures:** | | | |
| Mortality (in-hospital) | 12* (9.30%) | 39 (4.38%) | 28* (7.63%) |
| Mortality (three-year follow-up) | 24 (31.2%) | 99 (24.1%) | 50 (30.9%) |
| LOS (Hospital) [d] | 14.0 [9.00;23.0] | 14.0 [10.0;21.0] | 15.0 [10.0;24.0] |
| LOS (ICU) [d] | 6.00* [4.00;12.2] | 5.00 [3.00;9.00] | 6.00* [4.00;11.8] |
| Time of ventilation [h] | 8.00 [5.00;16.0] | 9.00 [6.00;17.0] | 12.0* [6.00;21.0] |
| KDIGO: |  |  | * |
| Stage 0 | 80 (66.7%) | 641 (77.0%) | 229 (68.2%) |
| Stage 1 | 17 (14.2%) | 89 (10.7%) | 35 (10.4%) |
| Stage 2 | 2 (1.67%) | 14 (1.68%) | 13 (3.87%) |
| Stage 3 | 21 (17.5%) | 89 (10.7%) | 59 (17.6%) |
| KDIGO Stages 1-3 | 40* (33.3%) | 192 (23.0%) | 107* (31.8%) |
| Incidence of hemodialysis | 22* (17.7%) | 90 (10.4%) | 59* (17.3%) |
| **Mean percentage of days on ICU without organ failure:** | | | |
| Respiration | 54.1%* | 65.5 | 63.8 |
| Liver | 96.5% | 97.0% | 96.4% |
| Circulation | 45.7%* | 61.0% | 53.3%* |
| Central nervous system | 78.8% | 81.1% | 79.5% |
| Kidneys | 82.2% | 86.9% | 83.3% |

Significant group differences are displayed as asterisks in columns for groups L and H with reference to group N. Renal dysfunction was assessed according to the KDIGO classification, calculating the change in serum creatinine from pre-op until POD7. Days of organ failure were calculated according to the criteria of the Sequential Organ Failure Assessment score (SOFA). Respiration: PaO2/FiO2<300; Bilirubin: ≥ 2.0 mg/dl; Circulation: Use of epinephrine or norepinephrine; Central nervous system: GCS<13; Kidneys: Serum creatinine ≥ 2.0 mg/dl.

Table S2c: Selected outcome parameters – CABG&Valves only

|  | Group L | Group N | Group H |
| --- | --- | --- | --- |
|  | N=80 | N=352 | N=106 |
| **General outcome measures:** | | | |
| Mortality (in-hospital) | 3 (3.75%) | 17 (4.83%) | 13* (12.3%) |
| Mortality (three-year follow-up) | 10 (21.7%) | 53 (26.6%) | 22 (36.1%) |
| LOS (Hospital) [d] | 16.0 [12.0;28.0] | 14.0 [11.0;22.0] | 18.0 [11.0;23.0] |
| LOS (ICU) [d] | 6.00 [4.00;11.0] | 6.00 [4.00;10.0] | 7.00 [4.00;11.0] |
| Time of ventilation [h] | 11.0 [8.00;18.5] | 11.0 [7.00;19.0] | 15.0 [10.0;27.0] |
| KDIGO: |  |  |  |
| Stage 0 | 54 (68.4%) | 235 (68.7%) | 50 (52.6%) |
| Stage 1 | 13 (16.5%) | 48 (14.0%) | 16 (16.8%) |
| Stage 2 | 2 (2.53%) | 11 (3.22%) | 4 (4.21%) |
| Stage 3 | 10 (12.7%) | 48 (14.0%) | 25 (26.3%) |
| KDIGO Stages 1-3 | 25 (31.6%) | 107 (31.3%) | 45* (47.4%) |
| Incidence of hemodialysis | 9 (11.4%) | 44 (12.7%) | 24* (24.5%) |
| **Mean percentage of days on ICU without organ failure:** | | | |
| Respiration | 60.8 (37.9) | 57.6 (35.7) | 55.3 (35.7) |
| Liver | 99.6 (3.10) | 99.0 (7.71) | 100 (0.00) |
| Circulation | 48.9 (40.3) | 48.0 (41.3) | 44.5 (39.4) |
| Central nervous system | 73.4 (35.4) | 77.9 (30.6) | 70.7 (32.5) |
| Kidneys | 83.7 (31.7) | 84.0 (30.9) | 75.2 (35.0) |

Significant group differences are displayed as asterisks in columns for groups L and H with reference to group N. Renal dysfunction was assessed according to the KDIGO classification, calculating the change in serum creatinine from pre-op until POD7. Days of organ failure were calculated according to the criteria of the Sequential Organ Failure Assessment score (SOFA). Respiration: PaO2/FiO2<300; Bilirubin: ≥ 2.0 mg/dl; Circulation: Use of epinephrine or norepinephrine; Central nervous system: GCS<13; Kidneys: Serum creatinine ≥ 2.0 mg/dl.
